# *Porphyromonas* *gingivalis* gingipains potentially affect MUC5AC gene expression and protein levels in respiratory epithelial cells

**DOI:** 10.1002/2211-5463.13066

**Published:** 2020-12-30

**Authors:** Chihiro Miya, Marni E. Cueno, Ryuta Suzuki, Shuichiro Maruoka, Yasuhiro Gon, Tadayoshi Kaneko, Yoshiyuki Yonehara, Kenichi Imai

**Affiliations:** ^1^ Department of Oral and Maxillofacial Surgery II Nihon University School of Dentistry Tokyo Japan; ^2^ Department of Microbiology Nihon University School of Dentistry Tokyo Japan; ^3^ Department of Internal Medicine Nihon University School of Medicine Tokyo Japan

**Keywords:** epithelial cell, gingipains, immortalized cells, MUC5AC, *Porphyromonas gingivalis*, primary bronchial cells

## Abstract

*Porphyromonas gingivalis* (Pg) is a periodontopathic pathogen that may affect MUC5AC‐related mucus hypersecretion along airway epithelial cells. Here, we attempted to establish whether Pg virulence factors (lipopolysaccharide, FimA fimbriae, gingipains) affect MUC5AC in immortalized and primary bronchial cells. We report that MUC5AC gene expression and protein levels are affected by Pg culture supernatant, but not by lipopolysaccharide or FimA fimbriae. Cells treated with either Pg single (Kgp or Rgp) or double (Kgp/Rgp) mutants had altered levels of MUC5AC gene expression and protein levels, and MUC5AC staining of double mutant‐treated mouse lung cells showed that MUC5AC protein levels were unaffected. Taken together, we propose that Pg gingipains may be the primary virulence factor that influences both MUC5AC gene expression and protein levels.

## Graphical abstract

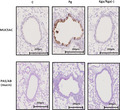

**Abbreviations**
COPD: chronic obstructive pulmonary disease
csp: culture supernatant
FimA: FimA fimbriae
KDP129: Kgp(–)
KDP133: Rgp(–)
KDP136: Kgp/Rgp(–)
Pg: *Porphyromonas gingivalis*

Periodontal diseases generally refer to disorders surrounding the teeth and could have developmental, genetic, inflammatory, metabolic, neoplastic, or traumatic origins [1]. Moreover, the causes of periodontal diseases have been linked to several factors, including host genetics, tobacco and alcohol use, HIV infection and AIDS development, improper nutrition, osteoporosis occurrence, diabetes development, stress exposure, impaired host immune response, and oral microorganisms present [2]. Among the many periodontal bacteria inhabiting the mouth, *Porphyromonas gingivalis* (Pg) has been implicated as a major agent involved in contributing to periodontal disease development and, likewise, progression [3, 4]. Pg is an oral Gram‐negative and a black‐pigmented anaerobic bacterium known to aggregate with other organisms within the subgingival plaque biofilm and, more importantly, invade sulcular gingival epithelial cells, which in turn result in the breakdown of epithelial cell junctions, thereby allowing the bacterium to enter deeply into periodontal tissues, and, consequently, colonize alveolar bone [3, 4, 5, 6]. Moreover, Pg possesses several potential virulence factors that allow it to evade host immune response, and these include cysteine proteinases, hemagglutinins, lipopolysaccharides, and fimbriae [7].

It has long been established that periodontal diseases contribute to certain systemic health problems and disease development, such as preterm birth and adverse events related to pregnancy, cardiovascular disease and stroke occurrence, diabetes onset, rheumatoid arthritis development, and pulmonary diseases [2, 8, 9, 10, 11, 12, 13]. In this regard, this would suggest that periodontal diseases (potentially ascribable to Pg) may have a role in the development of certain systemic diseases, in particular a pulmonary disease such as COPD (chronic obstructive pulmonary disease).

Under normal conditions, mucus clearance occurs in a host as a first line of defense against airborne pathogens and pollutants; however, under a pulmonary disease condition such as COPD, mucus hypersecretion occurs, thereby contributing to disease pathology and mortality [14]. Mucus is composed of 97% water and 3% solids, which in turn constitutes salts, lipids, polypeptides, cells, cell debris, and polypeptides (including mucins) [15, 16]. Among the solid components comprising mucus, mucins are glycoproteins that make up the majority [15]. Moreover, mucins that are expressed along the airway can be further grouped into membrane‐tethered and gel‐forming mucins [15, 17, 18]. In addition, among the numerous mucins found along airway epithelial cells, MUC5AC and MUC5B comprise approximately 90% of overall mucin content, and more importantly, MUC5AC expression and protein levels are high among COPD patients [18, 19, 20, 21]. In this regard, we hypothesize that Pg may influence both MUC5AC expression and protein levels in airway epithelial cells, thereby potentially contributing to COPD aggravation. However, to our knowledge, it is not clear which among the known Pg virulence factors could affect both MUC5AC gene expression and protein levels. A better understanding of the effects of Pg virulence factors on both MUC5AC gene expression and protein levels could lead to a putative correlation between oral and pulmonary diseases, which in turn may contribute to the development of new therapeutic approaches and diagnostic testing.

## Materials and methods

### Bacterial strains and culture

Pg strain ATCC 33277 was grown in brain–heart infusion (BHI) broth (Becton, Dickinson and Company, Sparks, MD, USA) supplemented with 5 µg·mL^−1^ hemin and 0.4 µg·mL^−1^ menadione. Bacterial cultures were grown in an anaerobic system (5% CO_2_, 10% H_2,_ and 85% N_2_ at 37 °C using a Model 1024 Anaerobic Chamber; Forma Scientific) for 48 h [22, 23]. Subsequently, culture supernatant (csp) was collected by centrifugation at 10 000 ***g*** at 4 °C for 20 min and filter‐sterilized through a 0.22‐µm pore size membrane filter to remove bacterial cells. Additionally, since medium components and salts were not removed, the same culture media (with no bacteria) were used for control treatments. Moreover, no additional standardization factor nor dilution procedure was performed in order to maintain raw data measurements produced during incubation, and similarly, in order to establish any data pattern, three independent samples were used [22, 23]. Csp pH was likewise determined and was found to be within pH 6.8–7.0. ATCC 33277‐derived mutant strains [KDP129 (*Δkgp*), KDP133 (*ΔrgpAΔrgpB*), and KDP136 (*ΔkgpΔrgpAΔrgpB*)] were kindly provided by Prof. K. Nakayama (Nagasaki University, Nagasaki, Japan) [24]. For this study, wild‐type strain ATCC 33277 and mutant strains (KDP129, KDP133, KDP136) were grown to late log phase, and thereafter, csp was collected. Moreover, KDP129 was designated as Kgp(–), KDP133 was designated as [Rgp(–)], and KDP136 was designated as [Kgp/Rgp(–)].

### Pg virulence factors

Representative Pg virulence factors used throughout this study were the lipopolysaccharide (LPS) at 100 ng·mL^−1^ (final concentration) and FimA fimbriae (FimA) at 1 µg·mL^−1^ (final concentration). Pg LPS was commercially obtained from InvivoGen (San Diego, CA, USA), whereas Pg FimA was kindly provided by Prof. Y. Hasegawa (Aichi Gakuin University, Aichi, Japan) [25]. Both Pg virulence factors were directly applied to downstream *in vitro* cell cultures.

Pg LPS and FimA final concentrations were patterned from published works unrelated to our group that showed similar LPS concentration used [26, 27] and, likewise, earlier works related to our group that showed comparable effects to Pg csp [22, 23]. Moreover, FimA was used for this study since it plays an important role in host adhesion [28] and its absence inhibited Pg mutants (without FimA) from invading human epithelial cells [29, 30].

### NCI‐H292 cell culture

NCI‐H292 human bronchial epithelial cell line was maintained in RPMI medium (Sigma‐Aldrich, St. Louis, MO, USA). Moreover, cell culture medium was supplemented with 10% fetal bovine serum, 100 U·mL^−1^ penicillin, and 100 µg·mL^−1^ streptomycin. Additionally, NCI‐H292 human bronchial epithelial cell was incubated in cell culture medium at 37 °C in a 5% CO_2_ atmosphere.

### Primary human epithelial cell culture

Commercially available primary human bronchial epithelial cells were cultured in bronchial epithelial cell medium with growth supplement following manufacturer’s recommendations. Primary cell, growth medium, and growth supplement were purchased from ScienCell Research Laboratories (Carlsbad, CA, USA). Briefly, primary cells were grown under the following conditions: 37 °C incubation temperature and 48‐h incubation time in a CO_2_ chamber. Cell growth and concentration were visually confirmed through a microscope and, likewise, via optical density.

### Quantifying *in vitro* MUC5AC gene expression

Cells were stimulated with medium containing Pg csp [2.5% (25 µL·mL^−1^); 5% (50 µL·mL^−1^); 10% (100 µL·mL^−1^), (v/v)] and incubated at 37 °C for 24 h. Total RNA was extracted using the RNeasy Plus Mini Kit (Qiagen, Hilden, Germany) according to the manufacturer’s instructions. cDNA synthesis from total RNA was performed with PrimeScript™ RT Master Mix (Takara Bio, Shiga, Japan). MUC5AC transcript levels were assessed using quantitative real‐time PCR (Takara Bio) that was performed using the SYBR Green method. Primer sequences used for the amplification of each gene were as follows: human MUC5AC, forward 5′‐GGA ACT GTG GGG ACA GCT CTT‐3′ and reverse 5′‐GTC ACA TTC CTC AGC GAG GTC‐3′; mouse MUC5AC, forward 5′‐CAT GGA GGG GAC CTG GAA AC‐3′ and reverse 5′‐CCA CAC TGG GGT CAC ACT TC‐3′; human glyceraldehyde 3 phosphate dehydrogenase (GAPDH), forward 5′‐ACC AGC CCC AGC AAG AGC ACA AG‐3′ and reverse 5′‐TTC AAG GGG TCT ACA TGG CAA CTG‐3′; and mouse β‐actin, forward 5′‐GGT CAG AAG GAC TCC TAT GTG G‐3′ and reverse 5′‐TGT CGT CCC AGT TGG TAA CA‐3′. PCR assays were performed using a TP‐800 Thermal Cycler Dice Real‐Time System (Takara Bio). These experiments were performed in triplicate. Calculated gene expression levels were normalized to either GAPDH or β‐actin mRNA levels.

### *In vitro* MUC5AC protein measurements

MUC5AC concentrations in the cell culture supernatants were measured using an ELISA Kit (Cloud‐Clone Corp, Minneapolis, TX, USA), according to the procedures recommended by the manufacturer. All experiments were performed in triplicate, and data are presented as the mean ± SD.

### Animal handling

All animal experiments were conducted in accordance with the Regulations and Guidelines on Scientific and Ethical Care and Use of Laboratory Animals of the Science Council of Japan, enforced on June 1, 2006. The study protocol was approved by the Institutional Animal Care and Committee of Nihon University School of Dentistry (Permit Number: AP16D047). Specific pathogen‐free male C57BL/6JJcl mice aged 7 weeks were obtained from CLEA Japan, Inc. (Tokyo, Japan). Mice were housed under standard conditions within the animal care facility at the Nihon University School of Dentistry, Tokyo, Japan. All mice were anesthetized with aspirating isoflurane and directly inoculated (via pipettor) with 50 µL of either Pg or Kgp/Rgp(–) mutant csp into the trachea once a day for 7 days. Pg csp volume used for *in vivo* experimentation followed the same conditions done in *in vitro* experimentation.

### Histological analysis and *in vivo* MUC5AC gene expression measurement

PBS‐treated mice (control) and Pg csp‐treated mice were sacrificed on Day 8. Lungs were surgically removed and divided into two with each lung fixed in 10% formalin, paraffinized, cut into sections of 6 µm thickness, and mounted onto glass slides. For immunohistochemical analysis, lung tissue slides were incubated with MUC5AC antibody (1:50; Abcam, Cambridge, MA, USA) overnight. Samples were then washed and incubated with secondary antibody (HRP‐conjugated goat anti‐rabbit antibody) for 30 min. Immunolabeling was visualized using 3,3′‐diaminobenzidine substrate, and the staining reaction was observed with a light microscope. Assessing mucin product from both the midsagittal section of the lungs and lung epithelial goblet cell hyperplasia, slides were subjected to periodic acid–Schiff (PAS) and Alcian blue (AB) staining in order to differentiate between acidic (blue), neutral (magenta), and mixtures of acidic and neutral (blue/purple) mucins. Additionally, mucin expression was evaluated under a light microscope. The measurement of *in vivo* MUC5AC gene expression was done through RT–qPCR assay using lung tissue. Briefly, lungs were homogenized using a BioMasher (Nippi, Tokyo, Japan) and centrifuged. After collecting the homogenized lung supernatant, total RNA was similarly extracted and RT–qPCR assay was performed as earlier described.

### Statistical analyses

All experiments utilized at least three independent samples (*n* = 3) and all measurements are presented as mean ± SD. Statistical significance of differences was further elucidated using *t*‐test, whereas significance level of 95% (*P* < 0.05) was considered statistically significant.

## Results

### Pg csp affects MUC5AC gene expression

**Fig. 1:**
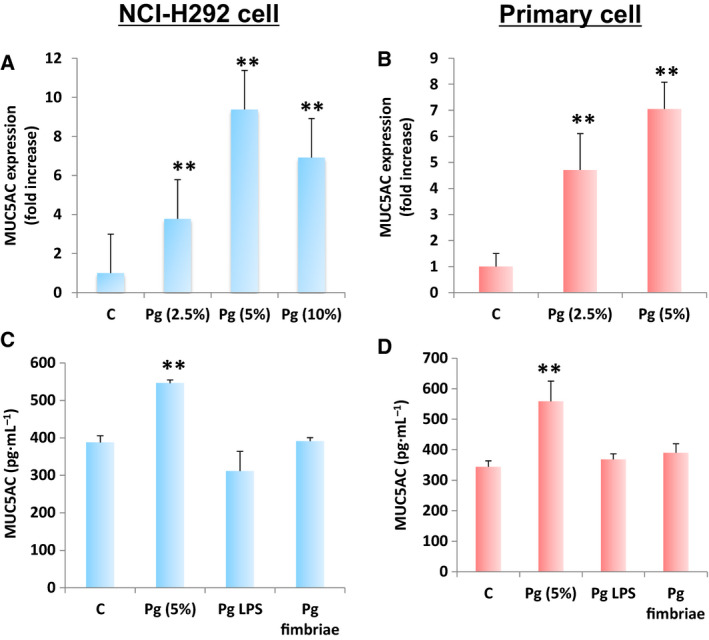
*Porphyromonas gingivalis* LPS and FimA do not affect *in vitro* MUC5AC gene expression and protein levels. MUC5AC gene expression in (A) NCI‐H292 and (B) primary bronchial cells is shown. Varying *P. gingivalis* (Pg) culture supernatant concentrations are indicated. MUC5AC protein levels in (C) NCI‐H292 and (D) primary bronchial cells are shown. Pg virulence factors [LPS (100 ng·mL^−1^) and FimA (1µg·mL^−1^)] are indicated. Negative [C] and positive [Pg csp (5%)] controls are assigned. Results presented are mean ± SD utilizing 3 independent samples. Statistical analyses were performed using *t*‐test (**P < 0.01).

Immortalized cell lines have a tendency to genetically and phenotypically differ from their tissue origin, while primary cells preserve many key markers and functions seen when using an *in vivo* model [31, 32]. In addition, when dealing with *in vitro* studies related to airway pathophysiology, it was recommended to utilize primary cells due to its numerous advantages [33]. To concurrently optimize the ideal Pg csp concentration that would affect MUC5AC gene expression and, likewise, establish whether MUC5AC gene expression is affected in both immortalized and primary cells, we independently incubated NCI‐H292 (immortalized) and primary cells with varying Pg csp concentrations and, subsequently, performed RT–qPCR. We initially used NCI‐H292 cells before utilizing primary cells in establishing the optimal Pg csp concentration due to the relative ease of growing immortalized cells over primary cells [34]. As seen in Fig. 1A, we found that Pg csp (regardless of concentration) affected MUC5AC gene expression, and more importantly, we established that Pg csp (5%) concentration is the ideal threshold in which MUC5AC gene expression is affected. This was consistent when using primary cells (Fig. 1B). We think that Pg csp concentrations above the threshold (5%) could be detrimental since Pg induces inflammation, which in turn may lead to cell death signaling [2, 35], thereby resulting in a decrease in MUC5AC gene expression. In this regard, Pg csp (5%) concentration was used in further downstream experimentations.

### Pg LPS and FimA putatively do not affect MUC5AC protein levels

Both Pg LPS and FimA are found on the outer surfaces and promote bacterial attachment to host cells, making these two bacterial components important virulence factors [35, 36, 37]. We performed ELISA to simultaneously determine and differentiate the effects of Pg (5%) concentration, Pg LPS, and Pg FimA on MUC5AC protein levels. As shown in Fig. 1C,D, Pg csp (5%) concentration was able to induce MUC5AC protein levels in both NCI‐H292 and primary cells, respectively. This is consistent with MUC5AC gene expression (Fig. 1A,B). However, both Pg LPS and FimA were found to putatively have no significant change in MUC5AC protein levels in both NCI‐H292 and primary cells (Fig. 1C,D), which could insinuate that neither Pg LPS nor Pg FimA would affect MUC5AC protein levels. Pg LPS causes the deregulation of the host innate immune system and the Pg LPS structure likewise changes according to the microenvironmental condition, whereas Pg FimA contributes to host inflammatory reactions and adheres to several oral substrates and molecules, such as extracellular matrix proteins, oral epithelial cells, and commensal bacteria [35]. Considering MUC5AC glycoproteins are produced by goblet cells within airway epithelial cells and pathologic airway epithelial remodeling results in increased goblet cell number accompanied by alterations in both stored and secreted mucins [38], we suspect that neither Pg LPS nor Pg FimA was adequate enough to force any pathologic airway epithelial remodeling, thereby resulting in unchanged goblet cell number, and thus, both MUC5AC gene expression and protein levels were unaffected.

### Pg gingipains influence MUC5AC gene expression and protein levels

**Fig. 2:**
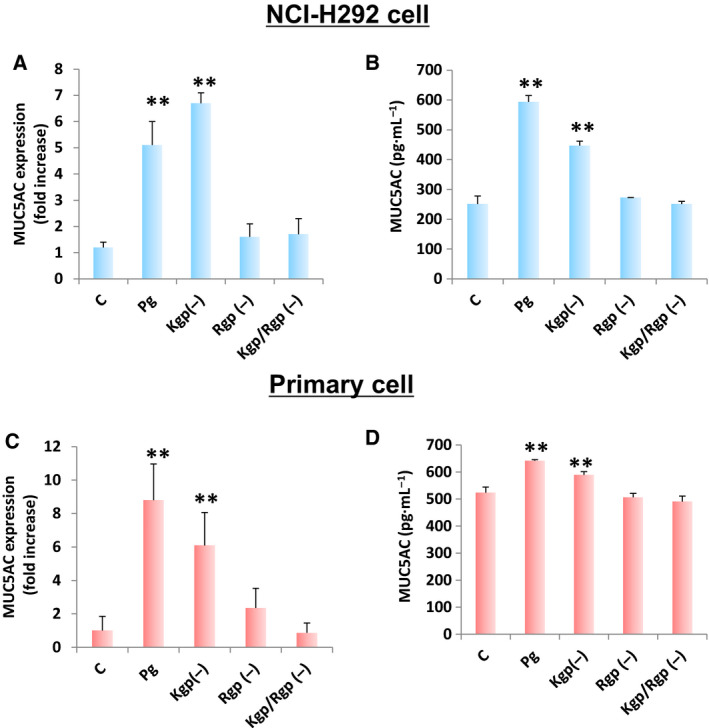
*Porphyromonas gingivalis* gingipains (Kgp and Rgp) putatively stimulate *in vitro* MUC5AC gene expression and protein levels. MUC5AC (A) gene expression and (B) protein levels in NCI‐H292 cells are shown. MUC5AC (C) gene expression and (D) protein levels in primary bronchial cells are designated. Negative [C] and positive [Pg] controls are assigned. *P. gingivalis* mutant forms [Kgp(–), Rgp(–), Kgp/Rgp(–)] are indicated. All Pg csp used (both wild‐type and mutants) for incubation had a final 5% concentration. Results presented are mean ± SD utilizing 3 independent samples. Statistical analyses were performed using *t*‐test (**P < 0.01).

Pg strains are capable of secreting numerous enzymes and metabolites, which likewise contribute to bacterial virulence [35]. In particular, Pg gingipains are a type of protease enzyme that accounts for 85% of extracellular proteolytic activity at the site of infection [39] leading to multiple infection events, including adhesion, hemagglutination, hemolysis, heme acquisition, degradation of host proteins, in housekeeping for gingipain maturation, dysregulation of host response, and tissue matrix destruction [40]. There are two types of gingipains [gingipain R (RgpA and RgpB) and gingipain K (Kgp)], and both are vital for the processing and maturation of Pg FimA and host tissue colonization [41, 42]. To elucidate whether Pg gingipain could affect both MUC5AC gene expression and protein levels, real‐time PCR and ELISA were performed using both immortalized and primary cells treated with either Pg wild‐type (Pg) or Pg mutants [Kgp(–), Rgp(–), Kgp/Rgp(–)]. We found that only Kgp(–)‐treated cells maintained significant difference (similar to Pg‐treated cells) in both MUC5AC gene expression and protein levels among immortalized (Fig. 2A,B) and primary (Fig. 2C,D) cells, respectively. Additionally, we observed that MUC5AC protein levels among Kgp(–)‐treated cells were higher compared with the control, but lower compared with Pg‐treated cells (Fig. 2B,D). In contrast, no significant difference in MUC5AC gene expression and protein levels was found between control and both Rgp(–)‐treated and Kgp/Rgp(–)‐treated cells. Moreover, both MUC5AC gene expression (Fig. 2C) and protein levels (Fig. 2D) in primary cells followed the same data pattern. Taken together, these results would suggest that (a) among Kgp(–)‐treated cells, MUC5AC gene expression and protein levels were partially affected, whereas (b) among Rgp(–)‐treated and Kgp/Rgp(–)‐treated cells, MUC5AC gene expression and protein levels were wholly affected. This would indicate that both Pg gingipains (Kgp and Rgp) could putatively affect MUC5AC gene expression and protein.

It is worth mentioning that Pg‐treated immortalized and primary cells differ in MUC5AC gene expression (Fig. 2A,C). We attributed this observation to the tendency of NCI‐H292 cells to have differential gene expression profiles compared with human epithelial cells [43]. This would further emphasize the difference between using immortalized and primary cells when performing *in vitro* experimentation related to airway pathophysiology [33]. Moreover, we likewise postulate that the difference between Kgp and Rgp with regard to MUC5AC gene expression and protein level measurements is ascribable to Rgp catalytic activities having an influence on Kgp processing [42]. In this regard, we suspect that independently Kgp and Rgp would have partial effects on MUC5AC gene expression and protein levels. However, considering Rgp can affect Kgp, we further hypothesize that Rgp (asides from a direct effect on MUC5AC) would likewise have an indirect effect via Kgp. In a possible future work, it would be interesting to elucidate this hypothesis. Additionally, in line with our earlier suspicion, we used the Kgp/Rgp(–) mutant in further downstream experimentations.

### Simulated Pg aspiration in mice lung‐induced MUC5AC gene expression and stimulated histochemically detectable MUC5AC and mucin

**Fig. 3:**
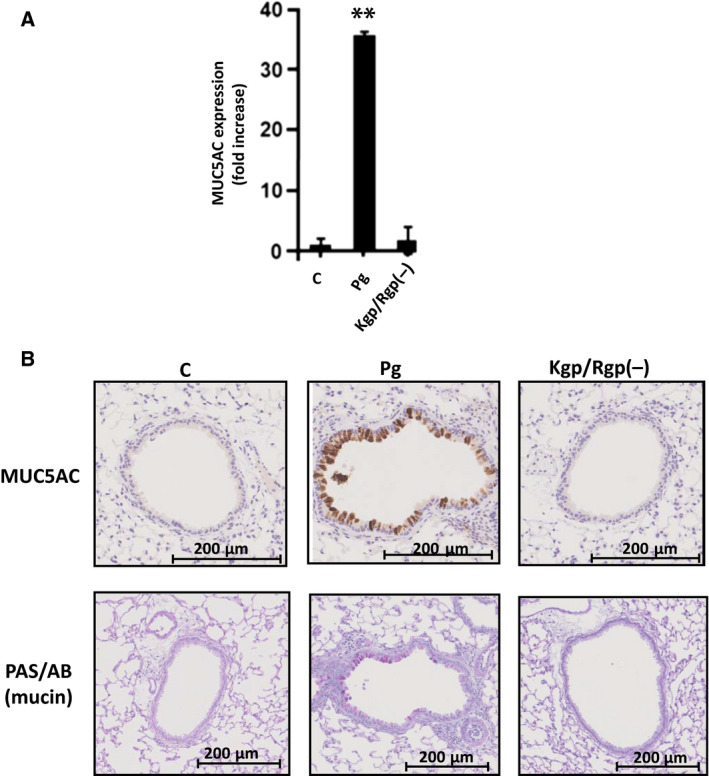
*Porphyromonas gingivalis* potentially induce *in vivo* MUC5AC gene expression and stimulate histochemically detectable MUC5AC and mucin along airway epithelial cells. (A) MUC5AC gene expression by RT–qPCR assay. Mice lung tissue treated with control, *P. gingivalis* wild‐type, and Kgp/Rgp(–) mutant is indicated. MUC5AC fold increase is shown. Results presented are mean ± SD utilizing 5 independent C57BL/6JJcl mice. Statistical analyses were performed using t‐test (**P < 0.01). (B) Histochemical staining of *in vivo* MUC5AC and PAS/AB (mucin). MUC5AC (brown‐stained cells) and mucin (purple‐stained cells) are shown. Mice lung tissue treated with control, *P. gingivalis* wild‐type, and Kgp/Rgp(–) mutant is indicated. Histochemical stains of MUC5AC and PAS/AB (mucin) are specified.

Basic studies related to airway pathophysiology have primarily relied on using cell lines and performing *in vitro* experimentation; however, result translatability in an *in vivo* situation has always been the ultimate goal [44]. To further verify the effects of Pg csp on lung MUC5AC, both Pg wild‐type and mutant [Kgp/Rgp(–)] were independently used to simulate aspiration in mice lungs, and subsequently, lung MUC5AC gene expression and histochemical staining of both MUC5AC and mucin were performed for confirmation. We found that lung MUC5AC gene expression was increased among Pg‐treated mice lungs compared with both control and Kgp/Rgp(–) mutant (Fig. 3A). Moreover, as shown in histochemical staining (Fig. 3B, *upper panel*), MUC5AC protein accumulated along airway epithelial cells of Pg‐treated mice, whereas no MUC5AC protein accumulation occurred in both control and Kgp/Rgp(–) mutant. Furthermore, these results are likewise consistent with mucin accumulation along airway epithelial cells of Pg‐treated mice (Fig. 3B, *lower panel*). Taken together, these results are consistent with our earlier results (Figs 1C,D and 2A–D), which would further imply that our *in vitro* results were translatable in an *in vivo* situation. More importantly, this would insinuate that Pg (through Pg gingipains) has the potential to affect respiratory epithelial cells.

It is worth mentioning that there is limited PAS/AB staining observed in Fig. 3B (lower panels) compared with MUC5AC staining (upper panels). We suspect that this could be ascribable to MUC5AC staining utilizing specific antibodies that are unaffected regardless of pH, whereas PAS/AB staining is affected by basic pH [45].

## Discussion

Pg plays a significant role in stimulating periodontal diseases [3, 4], whereas periodontal diseases have been postulated to contribute to pulmonary diseases [12]. Throughout this study, we attempted to establish the possible correlation between Pg‐associated periodontal diseases and mucus‐affected pulmonary diseases, such as COPD. In particular, we identified the major Pg virulence factor that putatively affected both MUC5AC gene expression and protein levels.

Periodontal diseases are characterized as complex, multifactorial, and polymicrobial infections mainly driven by periodontopathic bacteria (mostly Gram‐negative and anaerobic bacteria) growing along the gingival [2, 46]. In addition, periodontal diseases generally exhibit an inflammatory pathologic state along the gingiva and periodontium [47]. In most cases, periodontal diseases are associated with Pg, which in turn has several virulence factors that independently contribute to periodontal disease progression [35]. In this study, we were able to establish that among the common Pg virulence factors studied, both MUC5AC gene expression and protein levels are not affected by LPS and FimA, but are only affected by gingipain.

Pg LPS is a large molecule (10 kDa) that makes up the bacterial outer membrane and functions in disrupting innate host surveillance, inhibiting osteoblastic differentiation and mineralization within periodontal ligament stem cells [48, 49], whereas FimA is thin surface bacterial appendages protruding from the outer membrane and contribute to periodontal inflammatory reactions [50]. Both LPS and FimA are virulence factors that require cell‐to‐cell contact to initiate pathogenesis [35, 36, 37]. In this regard, we postulate that both immortalized and primary epithelial cells do not have the ideal attachment sites that would allow effective bacterial colonization, thereby leaving MUC5AC gene expression and protein levels putatively unaffected. This may likewise explain why only trace amounts of Pg are found along airway tissues from patients suffering from certain respiratory diseases [2, 12, 13].

On the other hand, Pg gingipains belong to the cysteine proteinase family (also known as trypsin‐like enzymes) that cleaves polypeptides at the C‐terminal region to either arginine (Rgp) or lysine (Kgp) and, likewise, degrade extracellular matrix proteins, activate host matrix metalloproteinases, inactivate plasma proteinase inhibitors, cleave surface receptors, and deregulate inflammatory responses [51, 52, 53]. Considering Pg gingipains are one of the secreted enzymes that come in close contact with host cells [54], we believe that both MUC5AC gene expression and protein levels were affected among *in vitro* (immortalized and primary) and *in vivo* (mice lung) cells since Pg gingipains have direct contact to host cells. In particular, we suspect two simultaneous gingipain‐related virulence activities: (a) either weakening or inhibition of host cell surface structures, thereby increasing cell accessibility [51, 52, 53]; and (b) stimulation of MUC5AC gene expression and, subsequently, protein levels as part of the innate immune response [55, 56]. This would highlight the possible effects of Pg gingipains in inducing MUC5AC gene expression and protein levels among respiratory epithelial cells.

It is worth mentioning that since periodontal diseases have been associated with COPD aggravation [12, 13], whereas COPD is linked to aspiration pneumonia occurrence [57], and similarly, Pg gingipains were found to stimulate aspiration pneumonia [11]. We postulate that Pg gingipains may play a significant role in aggravating COPD. Admittedly, more work is needed to further prove this point.

In summary, we established that gingipains (Kgp and Rgp) are putatively the main Pg virulence factor that could potentially affect both MUC5AC gene expression and protein levels. In a periodontal disease scenario, we propose the following possible virulence activities related to Pg‐linked MUC5AC stimulation: (a) Oral Pg secretes Kgp and Rgp, which in turn travels down to pulmonary tissues (such as the tracheal and bronchial tissues) and, consequently, induces MUC5AC among pulmonary epithelial cells; and (b) oral Pg (bacterial cell itself) weakly attaches along pulmonary tissues and, concurrently, secretes Pg gingipains, which may result in higher levels of MUC5AC stimulation.

## Conflict of interest

The authors declare no conflict of interest.

## Author contributions

KI and MEC conceived and designed experiments. CM and RS performed experiments. KI and MEC wrote the paper. SM, TG, TK, and YY contributed reagents, materials, and analytical tools and, likewise, reviewed drafts of the manuscript. KI conceived the study, designed the experiments, and authored or reviewed drafts of the article. All authors have read and agreed to the published version of the manuscript.

## Data Availability

Data will be available from the corresponding author upon reasonable request.
